# Synthesis of vinyl-based silica nanoparticles by sol–gel method and their influences on network microstructure and dynamic mechanical properties of nitrile rubber nanocomposites

**DOI:** 10.1038/s41598-022-19664-w

**Published:** 2022-09-10

**Authors:** Reza Ghamarpoor, Masoud Jamshidi

**Affiliations:** Constructional Polymers and Composites Research Laboratory, School of Chemical, Petroleum and Gas Engineering, Iran University of Science and Technology (IUST), Tehran, Iran

**Keywords:** Chemical engineering, Materials chemistry, Polymer chemistry, Surface chemistry

## Abstract

Non-agglomeration and dispersion of silica nanoparticles in polymers and their interfacial interactions to polymer matrix are the most important factors that influence nanocomposites performance. In this work, vinyltriethoxysilane (VTES) as a low VOC emission coupling agent was used for surface modification of silica nanoparticles to prepare better dispersion in nitrile rubber (NBR) and improve its interfacial interactions to silica nanoparticles. The results of X-ray photoelectron spectroscopy, thermogravimetric analysis and Fourier transform infra-red spectroscopy demonstrated successful attachment of VTES molecules on the surface of silica nanoparticles. Dispersion of the modified silica nanoparticles in NBR matrix was studied using field emission scanning electron microscopy and rubber process analysis. Results demonstrated that VTES significantly improved dispersion of nanoparticles in rubbery matrix. The bound rubber content showed that VTES effectively built a bridge between the silica nanoparticles and the rubber matrix that led to promising mechanical performances and strong interfacial interactions. Effect of nanoparticle content on the mechanical performances (static/dynamic) of the NBR was evaluated. It was found that higher modulus and reinforcement indices was obtained at 3 and 5 wt% of nanoparticles. Moreover, these composites had extremely low rolling resistance, the best wet skid resistance and the lowest Heat-Build up.

## Introduction

Nitrile rubber (NBR) is an oil resistant synthetic rubber manufactured by copolymerization of acrylonitrile and butadiene monomers^1,2^. The major applications of NBR are in fuel hoses, rollers, gaskets, gloves and other products in which oil resistance is required^3^. The global NBR market size was 1835.9 million USD and it is expected to reach to 2450 million USD by the end of 2025, with a growing rate of 4.2% during 2021–2025^4^. Generally, NBR is filled with reinforcing fillers to illustrate good mechanical properties^5–8^.

In tire industry, nano silica is highly used as reinforcing fillers in the rubber compounds to increase their mechanical properties (i.e. tensile strength, modulus, abrasion resistance) and reduce their rolling resistance^9–13^. However, due to presence of silanol groups (Si–OH) on the surface of silica nanoparticles (SNPs), they have tendency to aggregate in the rubber matrix^11,14,15^. This causes poor dispersion of silica nanoparticles in rubber matrix and significantly reduces the performance of the rubber^16,17^. To eliminate the problem, a few researches have been performed on surface modification of silica nanoparticles^18,19^. Surface modification of nano silica plays a key role in improving performance of rubbery nanocomposite^20^.

Silane coupling agents (SCAs) are of the best modifiers that could reduce the polarity of silica nanoparticles and improve their dispersion in rubber matrix^21,22^. SCAs could also increase the interfacial interactions between nano silica and rubber matrix^23^. Rubber composites filled with SCA-modified silica nanoparticles showed better dynamic mechanical and static properties^24,25^.

Among the silanes, mercaptopropyltriethoxysilane (MPTES), Bis-3Triethoxysilylpropyl disulfide (TESPD) and Bis-3Triethoxysilylpropyl tetrasulfide (TESPT) have been used more for surface modification of nano silica and increasing its interfacial interactions to rubbery matrices^26–28^. SCAs could react chemically to the silanol groups of nano silica to improve nanoparticles dispersion in rubber matrix^29–32^. However, it should be noted that SCAs cause increment in the volatile organic compounds (VOCs) that attracted attention of the international community to this issue^33^. On this basis, using a silane with lower VOC emission (due to having fewer alkoxy groups and no sulfide groups^34,35^) could help in solving the problem.

Cai et al.^36^ used methyltrimethoxysilane (MTMS), ethyltriethoxysilane (ETES) and 1H,1H,2H,2H-perfluorooctyltriethoxysilane (HFOTES) to enhance the hydrophobicity and oleophobicity of silica nanoparticles. Mahtabani et al.^37^ investigated the effect of surface modification of nano silica with 3 aminopropyltriethoxysilane (APTES) on charge trapping and transport of (PP)/(ethylene-octene) copolymer (EOC)/silica nano-dielectrics. For this purpose, they grafted APTES to nano silica to create amine functional groups on the surface of nanoparticles and alter interfacial interactions and electronic features to the silica–polymer interface. The nucleating ability and hence the crystallinity of the nano-dielectric was improved by surface modification of nano filler.

Gunji et al.^38^ used VTES and MPTES for surface modification of silica. They found that re-dispersion of dried powders into the solvent was low (1%) for MPTES and high (95%) for VTES modified silica. The re-dispersion of VTES-MPTES doubly-modified silica was considerably improved compared to MPTES modified silica. By incorporating the VTES-MPTES doubly-functionalized silica to tire rubber, the stress–strain curve was improved considerably.

Liu et al.^39^ modified the silica nanoparticles (SNPs) with Bis-triethoxysilylpropyl tetrasulfide (TESPT). They added the modified and unmodified nano silica to NBR and examined the dispersion of the nanoparticles. They found that TESPT acted as a “bridge” to form a chemical bond between nano silica and NBR molecular chains which significantly improved dispersion of nano silica particles in NBR matrix and so induced the best reinforcing effects to NBR. Kapgate et al.^40^ used silane grafted nano silica as reinforcing filler for nitrile rubber (NBR). Silica nanoparticles was synthesized by sol gel method and the surface of silica was modified with γ-mercaptopropyltrimethoxysilane (γ-MPS). The mechanical properties of NBR (i.e. stress–strain, moduli and tensile strength) was improved in the sample contained silane-treated nano silica.

Nano silica has been modified with different silanes for various applications, so far. However, few studies has been performed on grafting of vinyl silane as an environmental friendly and low VOC emission coupling agent^41^ on nano silica and its effects on filler dispersion and polymer-filler interfacial interactions and so on the static/dynamic mechanical and curing behaviors of the nanocomposite.

In this research, vinyltriethoxysilane (VTES) was grafted by sol–gel method on surface of silica nanoparticles to increase their interfacial interactions to NBR matrix. The bonding of silane molecules to nanoparticles was investigated using FTIR, XPS, TG and HR-TEM analysis. The surface modified nanoparticles was applied at different concentrations to NBR compound. The effect of pure and silanized nanoparticles on curing, mechanical and thermomechanical properties of NBR was evaluated.

## Experimental

### Materials

The used polymer in this study was acrylonitrile butadiene rubber (NBR) with 33% acrylonitrile content and having Mooney viscosity of ML (1 + 4) at 100 °C and a specific gravity of about 0.98 approximately that was supplied from Kumho PolyCHEM. An industrial grade fumed silica nanoparticles (SNPs, 60.08 g/mol) were supplied from a local company. Absolute ethanol (46.07 g/mol, 99.98%, Merck Millipore, Iran), and ammonium hydroxide (35.04 g/mol, 28–30 wt% solutions of NH_3_ in water, Merck Millipore, Iran) were used for hydrolysis of silane molecules. Moreover, Triethoxyvinylsilane (190.31 g/mol, VTES, 97%, Sigma-Aldrich) was used for surface modification of SNPs. GA-120 (aromatic petroleum resin is a low molecular weight) as plasticizer was supplied from Rhine Tejarat Co (Iran). Tetramethyl thiuram disulfide (TMTD) (240.44 g/mol, 97%, Sigma-Aldrich, Germany) and *N*-cyclohexyl-2-benzothiazole sulfonamide (CBS) (264.409 g/mol, 99%, China) were used as curing accelerators in rubber compound. the used sulfur (S_x_, 32.065 g/mol), Carbon black (N660, 12.011 g/mol), zinc oxide (ZnO, 81.38 g/mol), stearic acid (SA, 284.48 g/mol), Paraffin wax (PW, 436.84 g/mol), dioctyl phthalate (DOP, 390.55 gr/mol), Calcium carbonate (CaCo_3_, 100.08 g/mol), toluene (92.14 g/mol), acetone (58.08 g/mol) were industrial grade and supplied from local companies and used as received.

### Synthesis of silica nanoparticles with VTES

**Figure 1:**
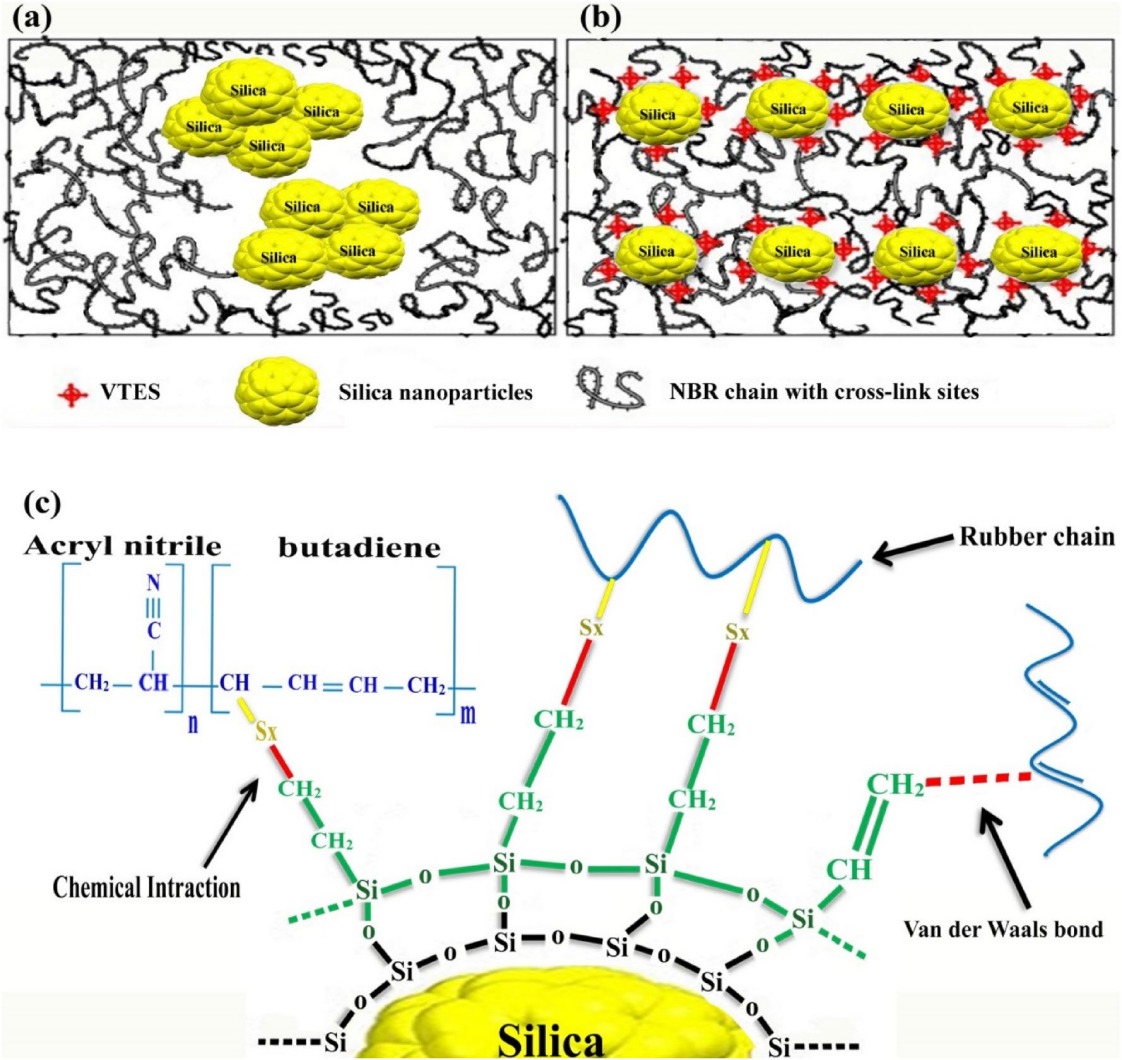
Interfacial interactions of VTES grafted nano silica to rubber matrix; (**a**) agglomeration of pure nano silica in NBR matrix, (**b**) dispersion of silanized nano silica in NBR matrix and (**c**) interfacial interfacial interactions between silanized nano silica and NBR.

Firstly (step 1), the silane (0.345 g) was added to 25 g of absolute ethanol and the process was followed by adding a 2 percent ammonia solution (NH_3_ + Water) (0.1 ml) to catalyze hydrolysis of the VTES. The solution was sonicated for 1 h in ultrasonic bath. Secondly (step 2), One g of nano silica was added to 50 g of absolute ethanol, then the mixture was sonicated for 1 h. Then, the prepared materials (i.e. step 1 and 2) were mixed. The reaction took place in a glass round bottom flask immersed in an oil heating bath and equipped with a mechanical stirrer (magnet), a cooler, and a thermometer. The mixture was stirred using a high-speed mixer at a speed of 1500 rpm for 4 h at 100 ^◦^C to achieve proper mixing. Then, the modified SNPs were extracted using a centrifuge at a speed of 3,000 rpm for 1 h and washed by mixture of absolute ethanol and acetone to remove non-reacted coupling agents which are attached by hydrogen bonding to the surface of grafted SNPs (G-SNP). Finally, they were dried in an oven at 80 °C for 12 h for condensation of silane molecules at the surface of SNPs. Figure 1 illustrates the mechanism of chemical bonding of VTES on SNP surface.

### Preparation of NBR nanocomposites

The used formulations for preparation of NBR compounds are demonstrated in Table 1. To prepare silica containing NBR compounds (R-NPs), the ingredients were mixed in an internal mixer (Banbury) at 90 °C. Silica nanoparticles were added to the NBR master-batch on a two-roll mill to prepare modified nanocomposites (R-GNPs). The compound was milled for 10 min at room temperature according to the ASTM D3182^42^. Then, for better nano filler dispersion, the nanocomposites were mixed in a HAAKE mixer for 10 min at rotating speed of 80 rpm and temperature of 80 °C. Increasing in the silica content during the mixing led to a significant increase in the viscosity and consequently increment in the mixture temperature^43^. After completing the mixing process, curing agent and accelerators were added to the masterbatch compounds at two roll mill. An MDR rheometer was utilized to determine the curing characteristics of the rubber compounds (at 160 °C). Aiming to prepare the samples for the tensile strength and hardness tests, the samples were vulcanized in a hot-press at 160 °C to form samples sheets.

**Table 1:** Formulation of NBR compounds at different content of SNPs.

Sample codes	R-Ctrl	R-NP1	R-NP2	R-NP3	R-NP5	R-NP10
Ingredients	Contents (phr)					
NBR (33%)	100	100	100	100	100	100
DOP	5	5	5	5	5	5
GA120	5	5	5	5	5	5
ZnO	5	5	5	5	5	5
Stearic acid	1	1	1	1	1	1
N660	63	63	63	63	63	63
CaCO_3_	33	33	33	33	33	33
PW	2	2	2	2	2	2
CBS	2	2	2	2	2	2
Sulfur	1.3	1.3	1.3	1.3	1.3	1.3
TMTD	0.6	0.6	0.6	0.6	0.6	0.6
Silica nanoparticle	0	2.179	4.358	6.537	10.895	21.79

Unit is in part per hundred parts of rubber (phr).

### Characterization methods

The grafting efficiency of silane molecules on nanoparticles surface were characterized using TGA thermo gravimeter (TG 209 F3, Tarsus-NETZSCH, Germany). Heating was carried out under a nitrogen atmosphere at rate of 10 °C/min from 40 to 600 °C. Fourier-transform infrared (FTIR) spectrometer (Bruker Optic GMBH, Germany) was used to characterize the chemical structure of SNPs and grafted nanoparticles (GNPs). X-ray photoelectron spectroscopy (XPS) (Thermo Electron Co, Waltham, USA) were used to determine compositions of chemical bonds in SNPs and GNPs. The microstructure of nanoparticles were studied by high resolution transmission electron microscope (HR-TEM) (FEI Tecnai F30 Twin 300 kV TEM).

Rheological behavior of the NBR samples was assessed using a Rubber Process Analyzer (RPA 2000, Alpha Technologies, USA) at frequency of 100 Hz and 0.5 degree angle^44^. The tensile strength test was carried out by an Instron Universal Testing Machine according to ASTM D 412^45^. The cure characteristics of the different rubbers were determined using a moving die rheometer (MDR, A0225-rheo Techpro, USA) at 160 °C according to ASTM D5289^44^. The morphology of nanocomposites were studied by field emission scanning electron microscope (FE-SEM). The elemental composition of the nanocomposites were analyzed by energy dispersive spectroscopy (EDS-mapping) using S-4700 microscope (Hitachi, Japan). The chemical structure of the nanocomposites was characterized using attenuated total reflectance Fourier-transform infrared spectroscopy (ATR-FTIR) (Spectrum 100, Perkin Elmer, Inc., USA). The glass transition temperature (T_g_) of the samples was measured using differential scanning calorimetry (DSC) (NETZCH STA 409 PC) under nitrogen atmosphere at a heating rate of 10 C/min.

### Calculations

The grafting ratio of VTES on SNPs was calculated based on the results of TGA analysis. The grafting ratio was calculated from the following equation:

1 $$ {\text{Grafting}}\;{\text{ratio}}\;\left( {{\text{R}}_{{\text{g}}} } \right) = { }\frac{{\left( {{\text{m}}_{2} - {\text{ m}}_{1} } \right){ } \times 100{\text{\% }}}}{{{\text{m}}_{1} }} $$

where m_1_ and m_2_ were the weight loss of the pure and modified nano silica, respectively.

The bound rubber content was measured for nanocomposites to study the interactions between VTES and SNPs. Firstly, the cured rubbers was cut into 1 cm^3^ cubes and wrapped in a copper mesh, then it was immersed in toluene for 2 days, Thereafter, the toluene was replaced with acetone for a day and then dried for 24 h. The bound rubber percentage was calculated from the following equation:

2 $$ {\text{Bound}}\;{\text{rubber}}\;{\text{content}} = \frac{{{\text{W}}_{3} - {\text{ W}}_{2} - {\text{ W}}_{1} { } \times \left( {1 -\upomega _{{\text{r}}} } \right)}}{{{\text{W}}_{1} -\upomega _{{\text{r}}} }}{ } \times 100{\text{\% }} $$

where W_1_, W_2_ and W_3_ were the weight of the rubber sample, the weight of the copper mesh, the weight of the copper packs that were dried, respectively. ω_r_ is the mass ratio of the matrix rubber calculated from the recipe of the compound.

Heat-Build up (HBU) and dynamic set were tested according to Goodrich Flexometer Model YS-III based on ISO 4666^46^. The samples were pressed on a load of 25 kg at 55 °C, and the frequency of pressing was 20 Hz. The dynamic set was calculated by the following formula:

3 $$ {\text{Dynamic}}\;{\text{set}} = \frac{{{\text{H}}_{0} - {\text{ H}}_{1} }}{{{\text{H}}_{0} }} \times 100{\text{\% }} $$

where H_0_ is the original specimen heights and H_1_ is the final specimen heights.

## Results and discussions

### Characterization of nanoparticles

The silica nanoparticles have tendency for agglomeration due to their high surface area and energy. The hydrogen bonding between surface hydroxyl groups lead to agglomeration of nanoparticles especially in polymeric matrices. In this work, VTES was utilized as coupling agent to prevent agglomeration of nanoparticles and improve their dispersion in NBR matrix.

**Figure 2:**
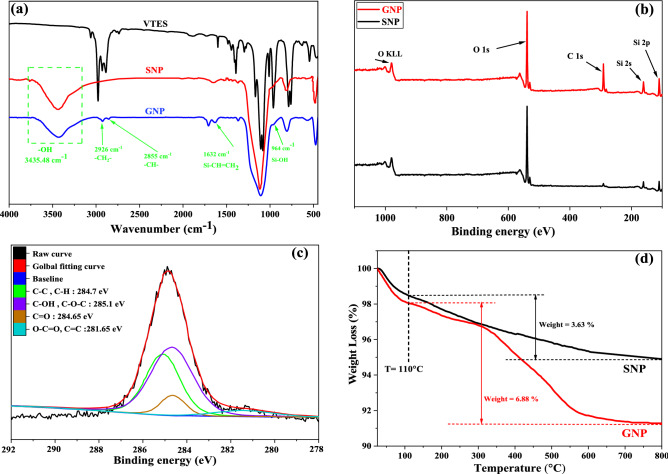
Surface modification of silica nanoparticles with VTES: (**a**) FT-IR curves, (**b**) wide-scan spectra of pure silica and modified silica (XPS), (**c**) C 1S spectrum of modified silica and (**d**) TGA analysis.

Figure 2a shows the FT-IR spectra of the pure and the surface modified silica nanoparticles. The FT-IR curve of VTES is also given for comparison. As can be seen, both the pure silica (SNP) and modified nanoparticles (GNP) showed strong peaks at 475, 806, and 1106 cm^−1^ that indicate asymmetrical, symmetrical and torsional vibrations of Si–O–Si bonds, respectively^47^. These bonds exist on the surfaces of both SNP and GNP and so they could not be used for proving VTES grafting on SNP. The observed peak at around 1632 cm^−1^ was related to absorption of –CH=CH_2_ bonds that did not exist in pure nano silica but appeared in GNPs due to grafting of VTES. The asymmetric vibrational peak of Si–OH was seen at 960 cm^−1^. Additionally, the absorption peaks of –CH_2_ and –CH, that related to VTES, was seen at 2926 and 2855 cm^−1^, respectively^48^. The broad and strong peak at wavelength of 3500 cm^−1^ was associated with the –OH stretching vibration. These OH groups were related to unreacted OH surface groups and absorbed humidity. It is clearly seen that the intensity of this peak decreased in GNPs. It was attributed to the reaction of hydrolyzed silane molecules to surface OH groups of nano silica that decreased their contents. Based on the results, it was found that VTES has been successfully grafted on SNPs.

Figure 2b,c presents the XPS spectra of the pure and modified silica nanoparticles. The peaks appeared at 284.5, 533.24, 155.92 and 104.07 eV was related to O 1s, C 1s, Si 2s, and Si 2p, respectively^49^. Based on the XPS results, the presence of O 1s, Si 2p, and Si 2s, which are the characteristic peaks of the silica was seen in both samples. However, the intensity of the peaks in the grafted silica was noticeably higher than the pure silica that was attributed to grafted silane molecules.

Figure 2c shows the high resolution C 1s spectra of the pure and modified silica nanoparticles. The presence of C 1s peak in the pure silica spectra (at a binding energy of 285.4 eV) was attributed to the –CH_3_ groups of graphite adhesive tapes used for holding of the samples. However, the deconvolution of the C 1s spectra for the grafted nano silica revealed that the binding energies of 281.65, 284.65, 285.1 and 284.7 eV could be attributed to C=C, C=O, C–OH/C–O–C and C–C/C–H bonds. Additionally, it was found out that the atomic percentage of Carbon in the grafted silica was higher than the pure silica that confirmed successful grafting of VTES.

Figure 2d presents the TGA results for pure and grafted nano silica and VTES. The declining trend in the weight loss of the pure nano silica in the first region (i.e. temperatures ranging from 25 to 110 °C) was attributed to the elimination of the absorbed water and low molecular weight materials (i.e. solvents). A slight decrease in the slope of the curve was seen in the second region (i.e. temperatures ranging from 110 to 800 °C) that was corresponded to the elimination of the surface hydroxyl groups.

The weight loss in the first region was higher for GNP than the pure nano silica that this was attributed to the absorbed humidity during surface modification process. The weight loss in the second temperature region was higher for GNP compared to SNP that this was corresponded to thermal degradation of grafted VTES molecules.

The grafting ratio (R_g_) of VTES on silica nanoparticle was calculated using TGA results. The calculated amount was 89% and indicated that most of the VTES molecules grafted on the nano silica surface.

**Figure 3:**
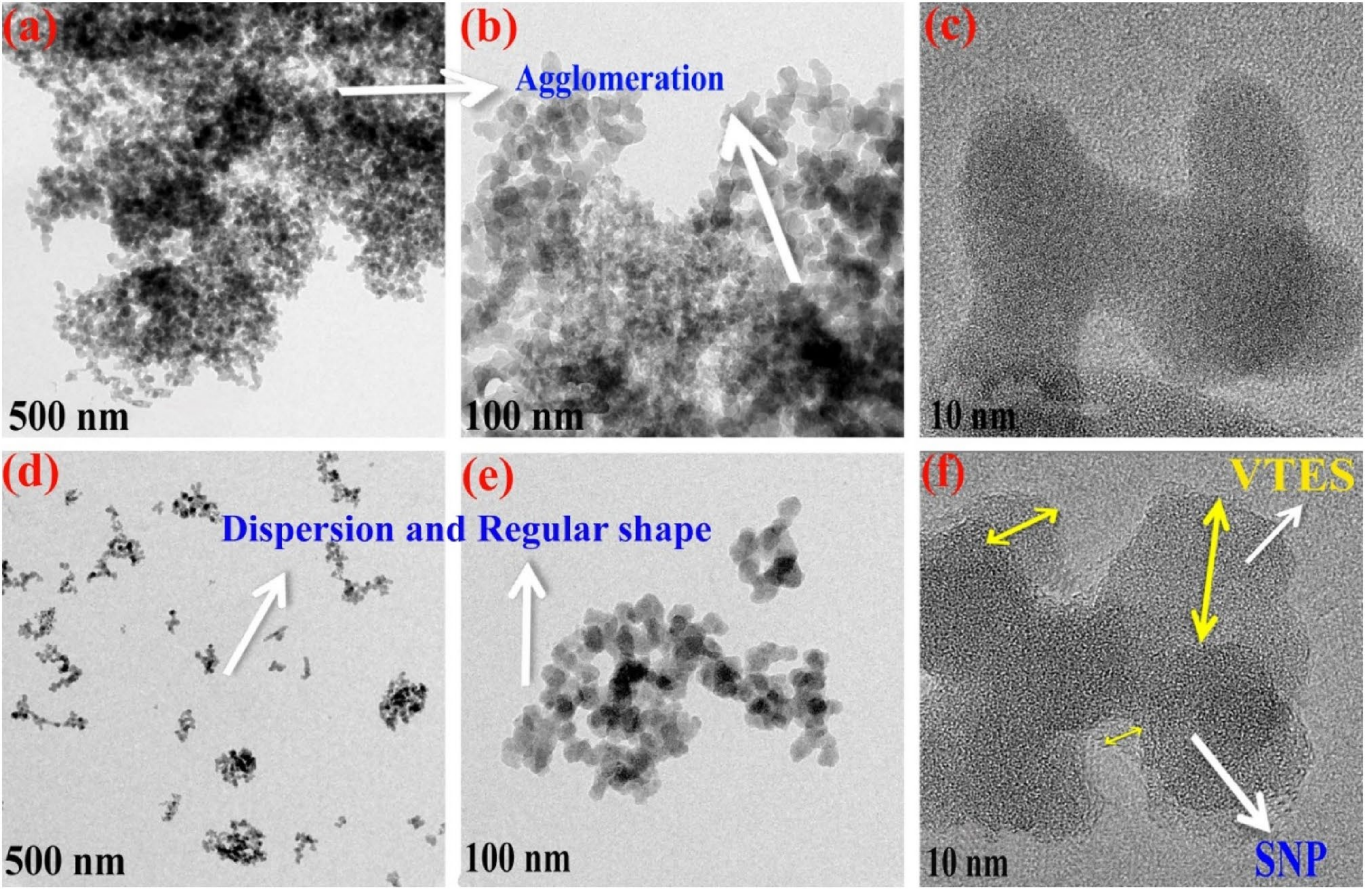
HR-TEM images of the silica nanoparticles; (**a**–**c**) Pure nano silica and (**d**–**f**) VTES grafted nano silica.

Figure 3 illustrates HR-TEM images of the pure and grafted nano silica. To illustrate the precise morphology of the samples, the images were taken at different magnifications. Obvious agglomeration could be observed in the pure silica nanoparticles (see Fig. 3a,b). Figure 3d,e clearly show that agglomeration decreased in the grafted silica nanoparticles due to steric stabilization that occurred by VTES^50,51^. The high resolution images of the samples confirmed grafting of VTES layer on silica nano particles (see Fig. 3c,f). The results proved formation of the chemical (covalent) bonds between silane molecules and nano silica surface.

### Effects of SNPs on NBR properties

**Figure 4:**
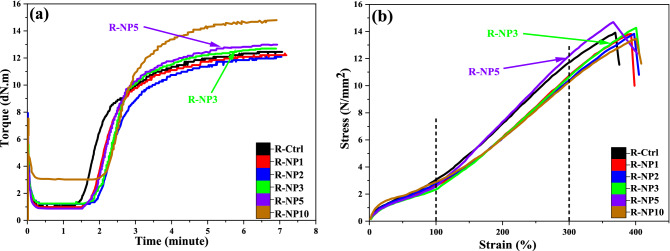
The curing and mechanical behaviors of the R-NP samples; (**a**) MDR results and (**b**) tensile test results.

Figure 4 and Table 2 illustrate curing and tensile properties of the NBR samples contained pure nano silica (i.e. R-NPs) at different concentrations of SNPs. Based on the MDR results, the M_H_ − M_L_ content which indicates crosslinking content changed in presence of the nano silica. An increment in the crosslinking was observed in the case of using more than 3 wt% of nano silica. It is well known that accelerators could be absorbed on the acidic surface of nano silica and this could cause increase in the curing time. However, by increasing in the nanoparticles content this problem was solved to some extent.

**Table 2:** The curing and mechanical properties of the pure nano silica-NBR samples.

Sample	R-ctrl	R-NP1	R-NP2	R-NP3	R-NP5	R-NP10
Cure time (t_90_, min)	4.145	4.35	4.432	4.324	3.961	4.42
∆M = M_H_ − M_L_ (dN m)	11.599	11.355	10.867	12.943	12.51	12.52
TS_2_ (min)	1.67	1.93	2.162	2.045	2.049	2.228
CRI	40.402	45.035	44.592	43.893	52.302	46.675
Tensile strength (MPa) (± SD)	13.91 ± 0.3	13.81 ± 0.5	13.83 ± 0.6	14.3 ± 0.65	14.7 ± 0.65	13.45 ± 0.7
Elongation at break (%) (± SD)	369.36 ± 3	393.07 ± 5	397.67 ± 5.7	400.64 ± 5.8	367 ± 5.7	400.8 ± 6.5
Modulus at 100% (MPa)	3.07	2.67	2.8	2.34	2.51	2.9
Modulus at 300% (MPa)	11.66	10.65	10.3	10.7	12.2	10.22
Reinforcement index	3.79	3.98	3.67	4.57	4.86	3.52
Hardness (Shore A)(± SD)	79 ± 0.5	75 ± 2	77 ± 2	78 ± 2	79 ± 2	81 ± 2

Considering the results, it was found that the best curing properties obtained at 5 wt% of pure nano silica. At higher nanoparticle contents (i.e. 10 wt.%), the M_H_ − M_L_ content decreased due to agglomeration of hydrophilic nanoparticles. On this basis, the nano silica contents of 3 and 5 wt% were selected as better nanoparticle concentrations and so, they were used for preparation of nanocomposites contained grafted nano silica (i.e. R-GNP samples).

Tc_90_ is one of the most important parameters that indicates time needed for 90% curing of the rubber sample. Results showed that the curing time decreased in samples contained 3 and 5 wt% of SNPs compared to Pristine NBR sample. This decrement in the curing time is desirable for rubber industry due to decrement in the consumed energy and production time of the rubbery parts.

T_S2_ content shows the scorch time of the compound. After scorch time, vulcanization process is initiated and crosslinks are generated. Results illustrated that the value of t_S2_ enhanced by increase in the nanoparticle content. The curing rate index (CRI) that indicates the slop of curing speed, was higher in the case of using 5 wt% of nano silica.

Considering the tensile test results, it was concluded that the highest tensile strength and elongation at break obtained at pure nano silica contents of 3 and 5 wt%. However, the amplitude of tensile strength was close to that of pristine NBR due to the poor dispersion of nanoparticles in the rubber matrix and their agglomeration. There were no significant changes in modulus at 100% of the nanocomposites, however, modulus at 300% of the nanocomposites decreased a little because of weak interfacial interactions between pure nano silica and polymer chains. Furthermore, incorporating the nanoparticles to NBR compound did not affect the hardness of nanocomposites especially at lower concentrations. Based on the results, it was generally found that pure nano silica did not improve the curing and mechanical properties of NBR.

### Effects of GNPs on NBR properties

The composition of nanocomposites incorporated grafted nano silica are shown in Table 3. As it is mentioned before, the R-GNP nanocomposites were prepared at nanoparticle concentrations of 3 and 5 wt% due to the obtained results for pure nano silica-NBR samples.

**Table 3:** The composition of R-GNP nanocomposites.

Ingredients	Sample code	
	R-GNP3 (phr)	R-GNP5 (phr)
Master batch	214	214
Curing System	3.9	3.9
GNPs^a^	6.8	11.32

^a^Unit is in parts per hundred parts of rubber (phr).

**Figure 5:**
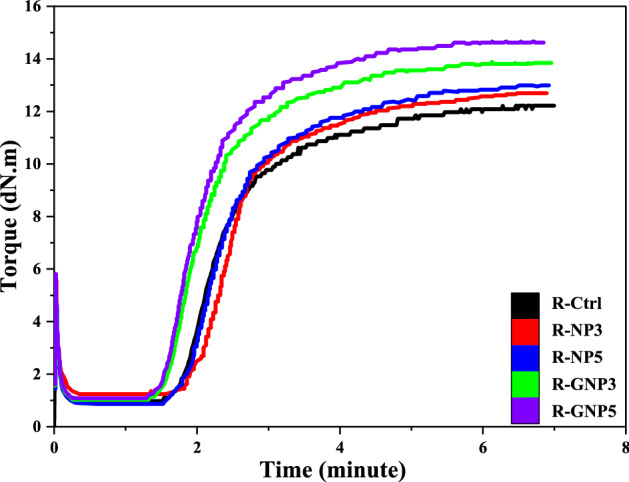
Comparative curing behaviors of the NBR nanocomposites at 3 and 5 wt% of pure and VTES grafted nano silica.

Figure 5 compares the curing characteristics of R-GNPs to R-NPs at nanoparticle contents of 3 and 5 wt%. It is clearly seen that by incorporating grafted nanoparticles to NBR compound the curing properties (i.e. crosslinking, cure time and CRI) improved significantly. This was attributed to the stronger interfacial interactions created between VTES grafted nano silica and rubber chains. After hydrolysis of VTES molecules and then condensation on nano silica surface, the hydrophilic nature of nano silica changed to superhydrophobic due to placing vinyl groups like a shell on the outer surface of nanoparticles. The presence of silane molecules eliminated absorbing accelerators on the acidic surface of nano silica that this improved curing rate of the nanocomposite. The optimum curing time (T_90_) decreased in the R-GNP samples compared to pure nano silica containing samples. Moreover, the M_H_-M_L_ and CRI values increased significantly in the case of using 5 wt% of grafted nanoparticles in NBR compound. This confirms co-vulcanization of grafted silane molecules with rubber chains that have been obvious in the case of using higher content of grafted nanoparticles (i.e. 5 wt%) in the rubber matrix. The lower cure time in R-GNP samples could be attributed to better dispersion of the nanoparticles in rubber matrix that improved heat transfer in the rubber matrix that helped homogeneous curing.

**Figure 6:**
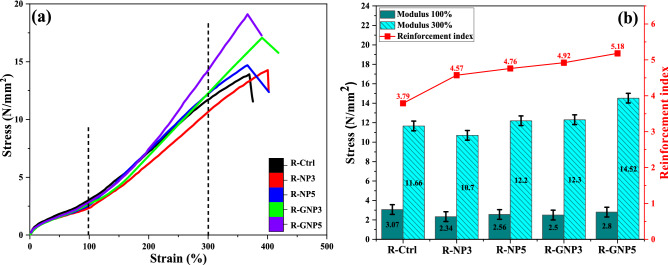
Comparative mechanical properties of the NBR nanocompoites at 3 and 5 wt% loading of nano silica; (**a**) tensile behaviour and (**b**) tensile test results.

Figure 6 illustrates comparative results of the mechanical performances of the NBR nanocomposites at 3 and 5 wt% of nanoparticles. The numerical data of the tensile strength and modulus at 100% and 300% of elongation are listed in Table 4. The results confirmed that silanization of nano silica caused significant improvement in the tensile strength, modulus at 300% strain and reinforcement index. The hardness was decreased by addition of grafted nanoparticles, due to oily nature of VTES, but the elongation at break was not affected.

**Table 4:** The curing and mechanical properties of R-GNP samples.

Properties	Sample code	
	R-GNP3	R-GNP5
Cure time (t_90_, min)	3.68	3.54
∆M = M_H_ − M_L_ (dN m)	12.82	13.53
TS_2_ (min)	1.78	1.72
CRI	52.63	54.94
Tensile strength (MPa) (± SD)	17.07 ± 0.5	19.1 ± 0.5
Elongation at break (%) (± SD)	390.79 ± 3.5	366.1 ± 3
Modulus at 100% (MPa)	2.5	2.8
Modulus at 300% (MPa)	12.3	14.52
Reinforcement index	4.92	5.18
Hardness (Shore A) (± SD)	74 ± 1	73 ± 1

### The bound rubber content

**Figure 7:**
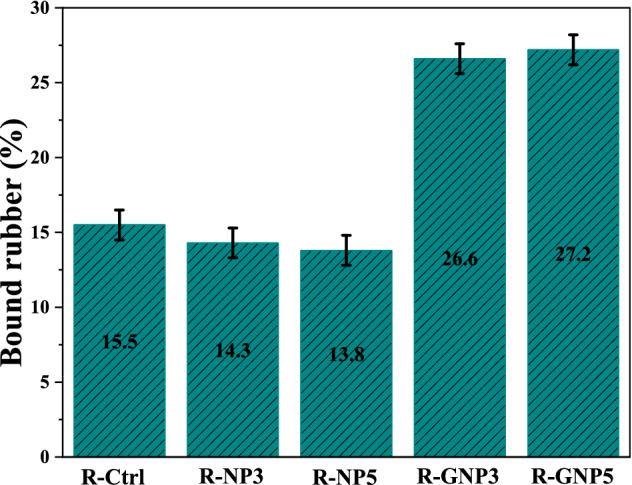
Total bound rubber contents of NBR based samples.

The bound rubber content could also be used to study the physical interfacial interactions between nano silica and the rubber matrix^52,53^. The bound rubber content was calculated for the NBR based samples that results are shown in Fig. 7. It was found that the bound rubber formation was not affected by addition of pure nano silica to NBR matrix. However, it was increased for the samples contained grafted nanoparticles. This confirms better diffusion of rubber in to the nano silica agglomerates due to better physical interaction between nanoparticles and rubber matrix. It also facilitates dispersion of nanoparticles that improves the mechanical properties.

### Characterization of the NBR samples

**Figure 8:**
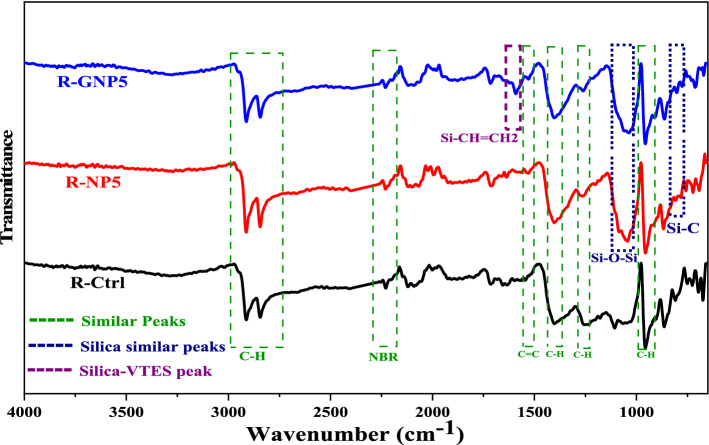
ATR-FTIR spectrums of NBR based compounds.

Surface of the NBR based samples were characterized using ATR-FTIR analysis that results are shown in Fig. 8. The absorption peak of nitrile group was seen at wavelength of 2228 cm^−1^ in the spectrum in all the samples^54^. This peak was affected by pure silica and silanized nanoparticles and shifted to 2229–2231 cm^−1^_,_ respectively. The peaks at 2844 and 2911 cm^−1^ were seen in all spectrums that should be related to C-H groups. The peaks at 1404 and 1530 cm^−1^ is associated with bending vibrations of –CH_2_ group. The overlapping peak at 1268 cm^−1^ is also related to bending vibration of CH_3_ group. The peak at 956 cm^−1^ was associated with bending vibrations of C–H in –CH=CH– group of NBR rubber that was seen in all the samples^55^. The presence of nanoparticles was confirmed by the peaks at 1108 and 1180 cm^−1^ (Si–O–Si group) in the nanocomposite samples. The peak at wavelength of 1530 cm^−1^ that was seen in the R-GNP5 sample was attributed to C=C bond of vinyl group in grafted silane. Furthermore, the peak at 806 cm^−1^ illustrates the tensile vibrations of Si–C bond in the grafted silane molecules that was not observed in the pristine NBR. The peak in 1602 cm^−1^ is related to –CH=CH_2_ group in VTES grafted nano silica containing NBR sample.

**Figure 9:**
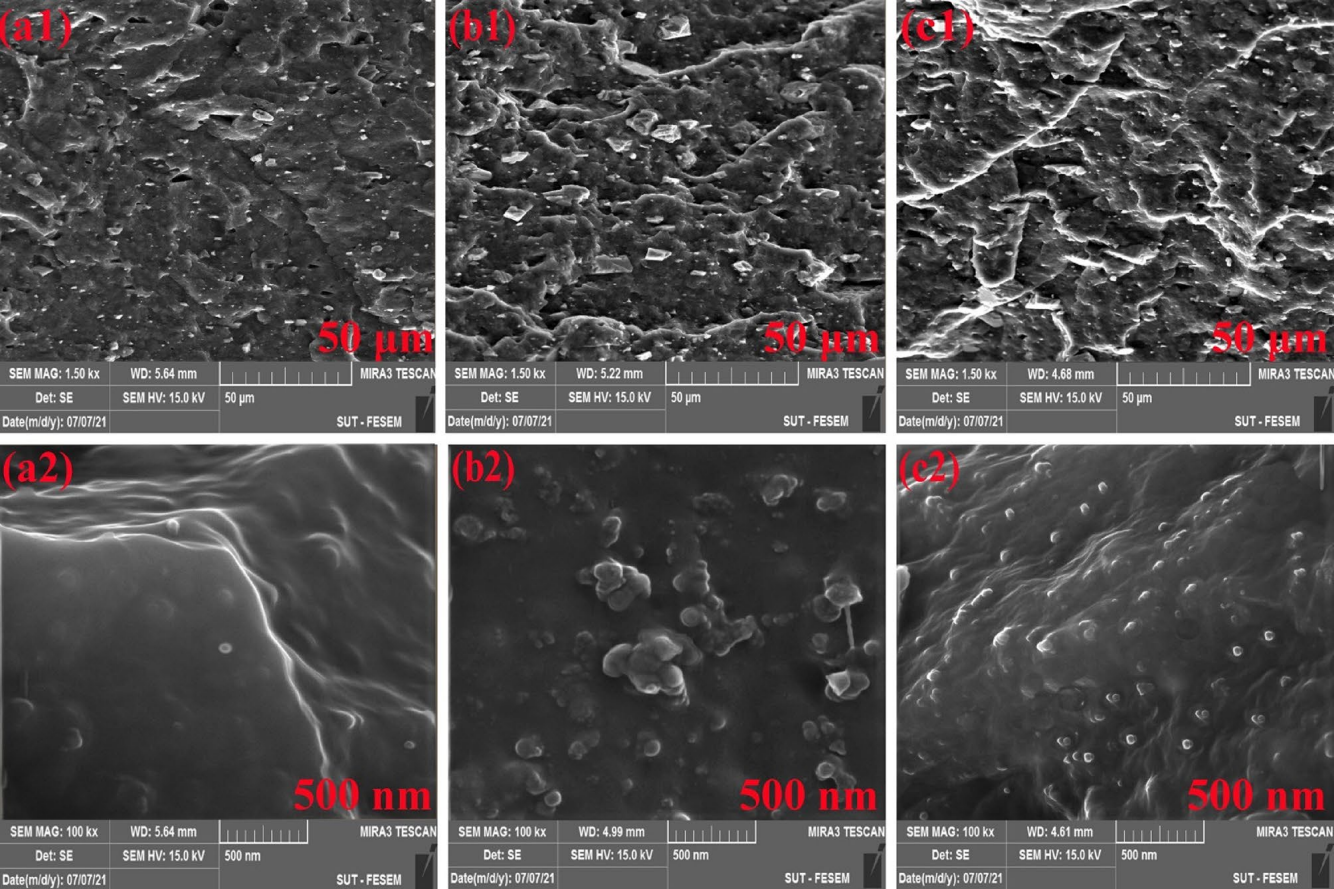
FE-SEM micrographs of NBR based samples; (**a1**, **a2**) R-ctrl, (**b1**, **b2**) R-NP and (**c1**, **c2**) R-GNP.

FE-SEM images from the fractured surface of NBR based samples were taken that results are shown in Fig. 9. According to Fig. 9a1–c1, it was found that roughness increased at the cross-section by using nano silica that shows effect of nanoparticles on improvement of toughness of the NBR matrix. However, the roughness was more in the sample contained grafted nanoparticles. Figure 9b2,c2 confirms the influence of silanization on better dispersion of the nanoparticles in NBR matrix that caused higher mechanical properties. In fact, the pure nanoparticles agglomerated due to presence of surface OH groups.

**Figure 10:**
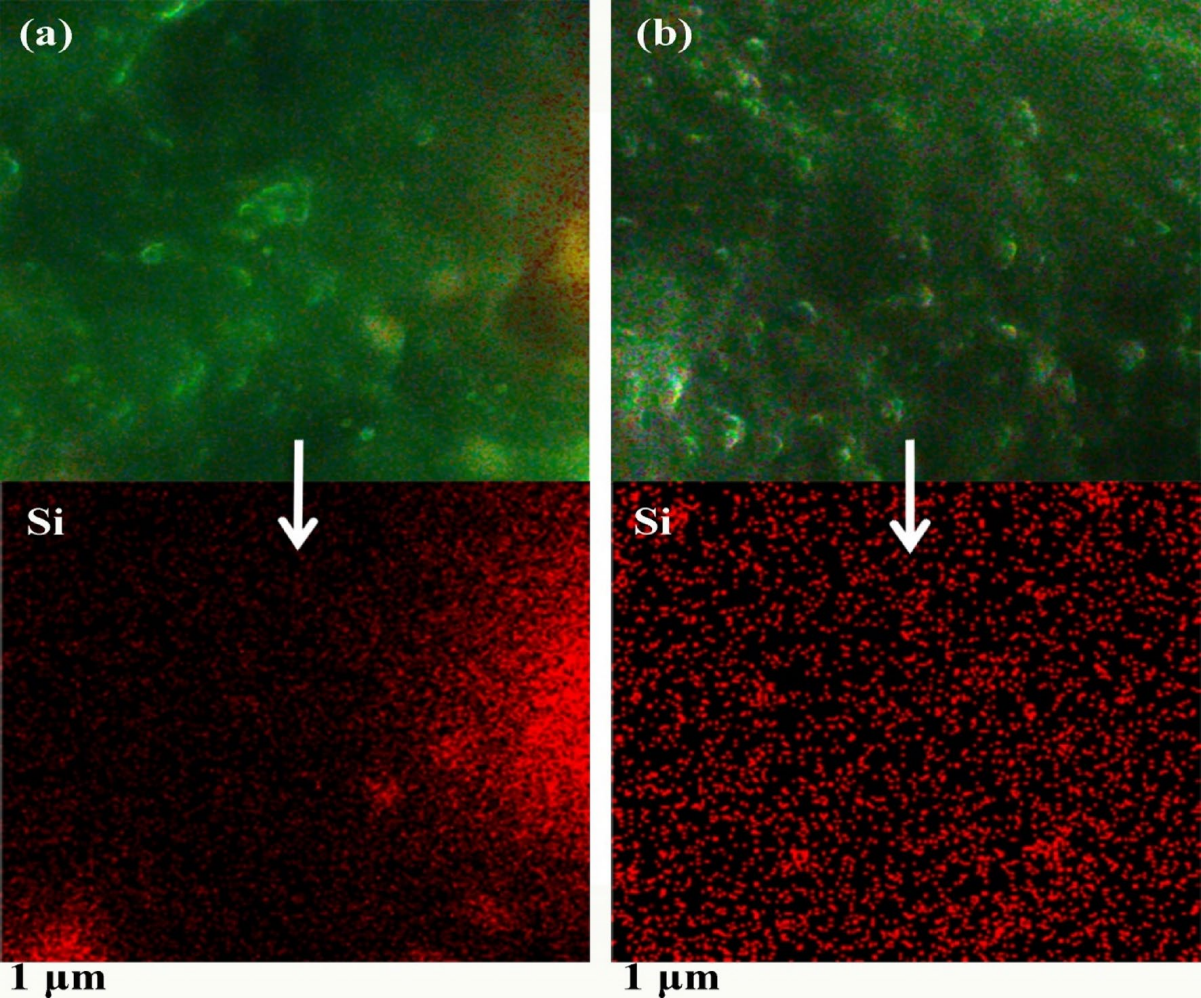
EDX-MAP Si elemental distribution images for: (**a**) R-NP, (**b**) R-GNP.

EDX mapping was used to identify the Si elemental composition of the composite (see Fig. 10). The elemental mapping of the sample showed a quite inhomogeneous and poor dispersion/distribution of Si element in the sample (see Fig. 10a). Figure 10b shows well dispersion of Si element in the rubber matrix. Homogeneous dispersion/distribution of the Si element was obviously obtained in the case of using VTES grafted nano silica.

### Payne effect

**Figure 11:**
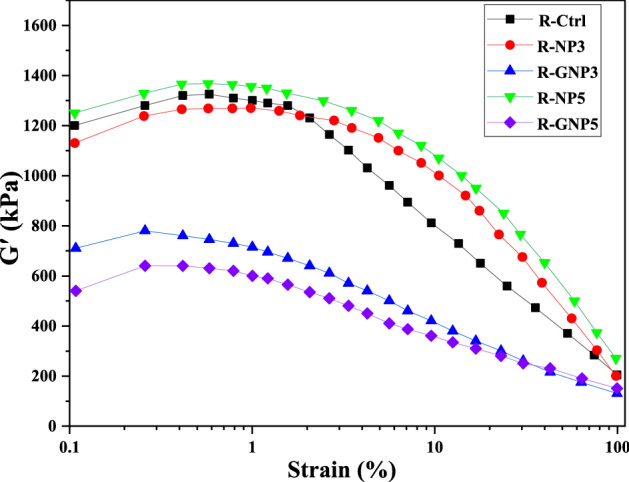
Storage modulus-strain curves of NBR based samples.

The decrease in the bulk modulus (G′) under high strains is called the Payne effect which shows the process ability of rubbery composites^18,56^. In general, the presence of carbon black in the control sample was the main factor that caused increment in the bulk modulus (see Fig. 11). However, the value of G′ instantly decreased after an increase in the strain that this could be explained by weak interfacial interactions between carbon black and the rubber matrix. In the rubber compounds that contained pure nano silica a high bulk modulus was obtained at low strains but the amount of G′ decreased slightly by increasing in the strain. In general, G′ and high Payne effect are associated with relatively poor dispersion of the filler in rubber matrix.

The results showed that by addition of 3 wt% of pure nano silica to NBR compound, G′ value decreased while it increased by addition of 5 wt% of nano filler. The highest value of G′ was obtained for the R-NP5 sample. According to the results, the amplitude of the strain experienced lesser slope that can be attributed to the filler-filler interactions in the compound. Interesting results was obtained in the case of using silanized nano silica in NBR compound. The bulk modulus decreased considerably at low and high strains at both nano silica contents. This event can be explained by the small VTES molecular chain that led to more energies absorbed between silica nanoparticles, since less Payne effect was obtained. The Payne effect result was consistent with results of FE-SEM analysis. On the other hand, chemical bonding of VTES with silica nanoparticles and also its reaction to the rubber –C=C– bonds led to an acceptable dispersion of modified nanoparticles in the rubber matrix.

### Differential scanning calorimetry (DSC) of the NBR samples

**Figure 12:**
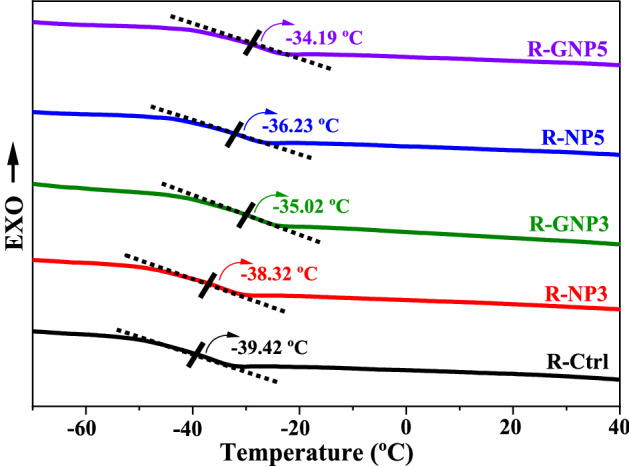
Glass transition temperature of the NBR samples based on DSC analysis.

The effect of chemical interactions between pure nano silica and silane grafted nano silica was evaluated by DSC analysis. The T_g_ values of the rubber samples briefly illustrated in Fig. 12.

The least value of T_g_ was obtained for the NBR control sample, while the maximum values was achieved in the case of using the silanized nano silica in NBR compound. Although pure nano silica could physically interact with polymer chains, but they also tend to aggregate and this decreases the filler’s effectiveness.

Based on the results, Tg increased by addition of nano silica to the NBR compound. In the case of using pure nano silica in NBR, this increment in Tg should be attributed to the effect of nanoparticles on limitations in movements of NBR chains. However, by silanization of the nano silica the interfacial interactions increased between nano silica and rubber chains that limited more the movements of the chains and increased Tg of the NBR.

### Dynamic mechanical performances of the NBR samples

**Figure 13:**
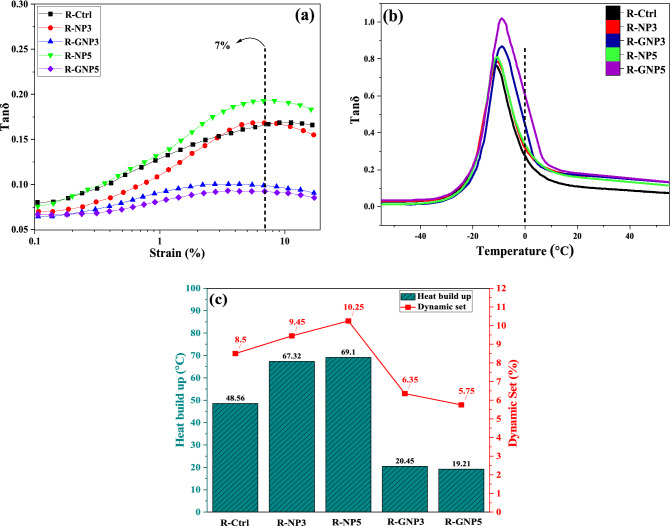
Dynamic mechanical performances of NBR/silica composites: (**a**) loss factor (tanδ-strain), (**b**) loss factor (tanδ-temperature) and (**c**) Heat buildup and dynamic set.

Figure 13 presents the RPA results. In general, it is believed that the loss factor tanδ obtained at 7% of strain (at 60 °C) could predict the rolling resistance of a rubber. According to Fig. 13a, the value of tanδ of the grafted nano silica containing NBR samples decreased in comparison to control sample and the samples contained pure nano silica. This event can be explained by the fact that in pure nano silica there are numerous hydroxyl groups that cause tendency of the nanoparticles to aggregate due to hydrogen bonding. However, in the grafted silica nanoparticles, the chemically bonded VTES molecules decrease the particle–particle interactions due to steric hindrance of silane molecules. The vinyl groups of VTES also shows good consistency to polymeric chains that this also helps to improve dispersion of the nanoparticles in NBR matrix. The lowest tanδ was observed in the NBR sample that contained 5 wt% of grafted nano silica.

The wet skid resistance of rubber is a very important factor which seriously affects the mechanical performance of O-Rings and Belts. The higher loss factor at 0 °C indicates the better wet skid resistance^57^. As shown in the Fig. 13b, the samples contained grafted nano silica illustrated the highest tanδ at 0 °C.

The heat-build-up test shows the compatibility of polymer chains and nano silica particles in rubbery material. Figure 13c shows that the highest temperature was observed in the compound contained pure nano silica. This was attributed to the aggregation of nano silica in rubber matrix. In contrast, nanocomposites prepared by grafted nano silica experienced the least increase in the temperature and dynamic set. This can be attributed to the suitable dispersion of nano silica particles in NBR matrix and decrement in the friction between filler-filler and matrix-filler.

## Conclusions

Silica nanoparticles were successfully modified by vinyltriethoxysilane (VTES). The grafting ratio of VTES on silica reached to 89%. VTES created strong chemical interactions between silica nanoparticles and NBR chains through double bonds and significantly improved the silica dispersion in the NBR matrix. Based on the results the following conclusions were obtained:

- Dispersion and interfacial interactions of silica nanoparticles to NBR matrix improved considerably by grafting of VTES on nano silica. This caused improvement in the curing and mechanical properties of the NBR nanocomposites.
- Grafting of vinyl silane on silica nanoparticles improved bound rubber content about twice times. This was occurred due to the formation of chemical bonds along with increased physical bonding at the rubber-filler interface.
- Based on the higher storage modulus of the nanocomposites at low and high strains, it was concluded that grafted vinyl silane on silica nanoparticles decreased filler-filler interactions and increased considerably filler-NBR interfacial interactions.
- Surface modification of silica nanoparticles by VTES caused up to 200 and 100% improvement in the rolling and skid resistance of the NBR nanocomposites.
- It was found that using 5 wt% of VTES grafted nano silica in NBR compound caused about 37 and 30% improvement in the tensile strength compared to pristine NBR and pure nano silica containing NBR. It also improved modulus of 300% about 25 and 19% compared to pristine NBR and pure nano silica containing NBR. VTES grafted nano silica also caused about 7% improvement in the reinforcing index of nanocomposite compared to pure nano silica at the same content.
- Surface modification of nano silica caused up to 72 and 44% decrement in the heat buildup and dynamic set of NBR compared to pure nano silica.
- Pure silica nanoparticles had not considerable effect on the mechanical properties of NBR and disturbed vulcanization of NBR. However, VTES as an environmental friendly silane decreased VOC emission as well as improving the mechanical and curing properties of NBR.

## Data Availability

All data generated or analysed during this study are included in this published article.
